# Magnetite Nanoparticles Enhancing H_2_‐Driven Biomethanation in a Mixed Microbial Community

**DOI:** 10.1002/gch2.202500367

**Published:** 2025-08-19

**Authors:** Matteo Tucci, Jasper I Sabangan, Carolina Cruz Viggi, Lucia Bertaccini, Francesca Iosi, Emilio D'Ugo, Daniela Uccelletti, Bruna Matturro, Andrea Firrincieli, Agnese Piacentini, Stefano Fazi, Federico Aulenta

**Affiliations:** ^1^ Water Research Institute (IRSA) National Research Council (CNR) Monterotondo 00015 Italy; ^2^ Core Facilities The Italian National Health Institute (ISS) Rome 00161 Italy; ^3^ Department of Infectious Diseases The Italian National Health Institute (ISS) Rome 00161 Italy; ^4^ Department of Biology and Biotechnology “C. Darwin” Sapienza University of Rome Rome 00185 Italy; ^5^ Department for Innovation in Biological Agro‐Food and Forest Systems University of Tuscia Viterbo 01100 Italy

**Keywords:** biogas upgrading, biomethanation, conductive nanomaterials, interspecies electron transfer, power‐to‐methane (P2M)

## Abstract

Biological methanation is increasingly considered for biogas upgrading. Here, the supplementation of conductive magnetite (Fe_3_O_4_) nanoparticles is investigated as a strategy to enhance H_2_‐driven biomethanation in a mixed hydrogenotrophic methanogenic community. An enrichment culture, maintained for over 180 days in a fill‐and‐draw anaerobic bioreactor under H_2_/CO_2_ feeding, is used to inoculate batch microcosms containing 0, 1.25, and 2.5 gFe L^−1^ of magnetite. Magnetite addition resulted in a dose‐dependent increase in maximum methane production rates—up to 13‐fold compared to controls—and sustained high hydrogen‐to‐methane conversion yields (78–107%). 16S rRNA gene sequencing reveals that archaeal community composition remained dominated by hydrogenotrophic *Methanobrevibacter* and *Methanobacterium* spp., whereas bacterial populations shifted from acetogenic *Sporomusa* and *Acetobacterium* spp. toward H_2_‐oxidizing *Paracoccus* and *Thauera* spp. at higher magnetite concentrations. Electron microscopy and energy‐dispersive X‑ray spectroscopy show that magnetite nanoparticles formed conductive networks bridging microbial cells, and fluorescence in situ hybridization confirmed co‐localization of methanogens and *Paracoccus* within these aggregates. The findings support a direct interspecies electron transfer (DIET) mechanism facilitated by magnetite, whereby *Paracoccus* spp. oxidize H_2_ and shuttle electrons to methanogens, accelerating biomethanation. These results highlight the potential of magnetite‐mediated DIET to improve power‐to‐methane processes and advance biogas upgrading technologies.

## Graphical abstract

Supplementation of magnetite nanoparticles to a mixed microbial culture accelerates H_2_/CO_2_ biomethanation by up to 13‐fold. The observed enhancement is accompanied by a marked enrichment of *Paracoccus* sp., suggesting the establishment of a mutualistic interaction with hydrogenotrophic methanogens via interspecies electron transfer.

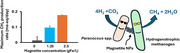

## 1 Introduction

In the context of the global energy transition, the development of carbon‐neutral and circular bioeconomy strategies is essential to achieve the decarbonization targets set by international climate agreements.^[^ ^1^ ^]^ Anaerobic digestion (AD) has emerged as a key technology in this framework, enabling the treatment of organic waste streams and the simultaneous production of biogas, a renewable energy vector predominantly composed of methane (50–70%) and carbon dioxide (30–50%).^[^ ^2^, ^3^ ^]^ Despite its advantages in terms of sustainability and waste valorization, raw biogas has a limited calorific value (≈21–25 MJ Nm^−^^3^) and cannot be directly injected into natural gas grids without prior purification. This has led to increasing interest in biogas upgrading technologies, aimed at enriching the methane content (>95%) and removing CO_2_ and other trace contaminants.^[^ ^4^ ^]^

Among the various upgrading strategies developed, biological methanation has gained traction due to its mild operational conditions, compatibility with AD, and ability to integrate with renewable electricity sources via power‐to‐methane (P2M) schemes.^[^ ^5^ ^]^ In H_2_‐driven biomethanation, molecular hydrogen produced by electrolysis is biologically converted to methane through the reduction of CO_2_, mediated by hydrogenotrophic methanogenic archaea. This process effectively upgrades biogas by reducing its CO_2_ content while simultaneously storing surplus renewable electricity in the form of CH_4_, a storable and transportable fuel.

Over the last years, P2M technologies have rapidly advanced reaching a Technology Readiness Level (TRL) of 7, with several systems now operating at demonstration or pre‐commercial scale. This accelerated progress has been driven in part by the involvement of companies such as Electrochaea GmbH, which have developed robust biomethanation processes based on hydrogenotrophic methanogens.^[^ ^6^, ^7^ ^]^

Despite of recent progresses, the practical implementation of H_2_‐driven still faces several engineering and microbiological challenges, including gas‐liquid mass transfer limitations, hydrogen diffusivity, and the inefficiency of microbial electron transfer mechanisms in complex microbial consortia.^[^ ^8^, ^9^ ^]^

In recent years, attention has increasingly turned to the role of conductive materials and nanomaterials in enhancing syntrophic interactions and overcoming some of these biological bottlenecks. In this context, the addition of conductive particles, such as magnetite, activated carbon, or biochar, has emerged as a promising strategy to enhance the anaerobic degradation of organic matter during methanogenesis.^[^ ^10^, ^11^, ^12^ ^]^ These materials facilitate direct interspecies electron transfer (DIET) between syntrophic bacteria and methanogenic archaea, bypassing the need for diffusible intermediates such as hydrogen or formate. This mechanism not only accelerates the overall conversion rate of complex organic substrates into methane but also improves process stability under high‐loading or inhibitory conditions. Furthermore, the presence of conductive particles can also promote the development of more electroactive microbial communities and reduce lag phases in anaerobic digestion systems. Overall, the integration of conductive materials has been reported to offer an effective means to boost the efficiency and resilience of methanogenic bioprocesses. Among currently studied (nano)particles, magnetite (Fe_3_O_4_) has emerged as a promising additive due to its high electrical conductivity and biocompatibility.^[^ ^13^ ^]^ Yet, while the role of magnetite in conventional AD and in the anaerobic oxidation of short‐chain fatty acids or other syntrophic processes has been explored,^[^ ^14^ ^]^ its influence on H_2_/CO_2_ biomethanation reaction has not been previously investigated.

A previous study,^[^ ^15^ ^]^ reported that a soluble redox mediator, anthraquinone‐2,6‐disulfonate (AQDS), could enhance the kinetics of H_2_/CO_2_ biomethanation in a mixed microbial community, most likely through a two‐step mechanism. In this mechanism, microbes rapidly used H_2_ to reduce AQDS into the highly soluble AH_2_QDS, which then served as a more efficient electron donor for the reduction of CO_2_ to methane. This study raised the intriguing hypothesis that interspecies electron transfer may enable metabolic reactions, even if typically carried out entirely by a single microorganism, to proceed more rapidly when shared by two (or more) cooperating partners. Building on this finding, the present study investigated whether H_2_‐dependent biomethanation could be facilitated by DIET via electrically conductive magnetite nanoparticles, offering a more direct mechanism compared to the use of diffusible redox mediators.

## 2 Results and Discussion

### 2.1 Characterization of the Hydrogenotrophic Methanogenic Culture Used in Biomethanation Tests

**Figure 1:**
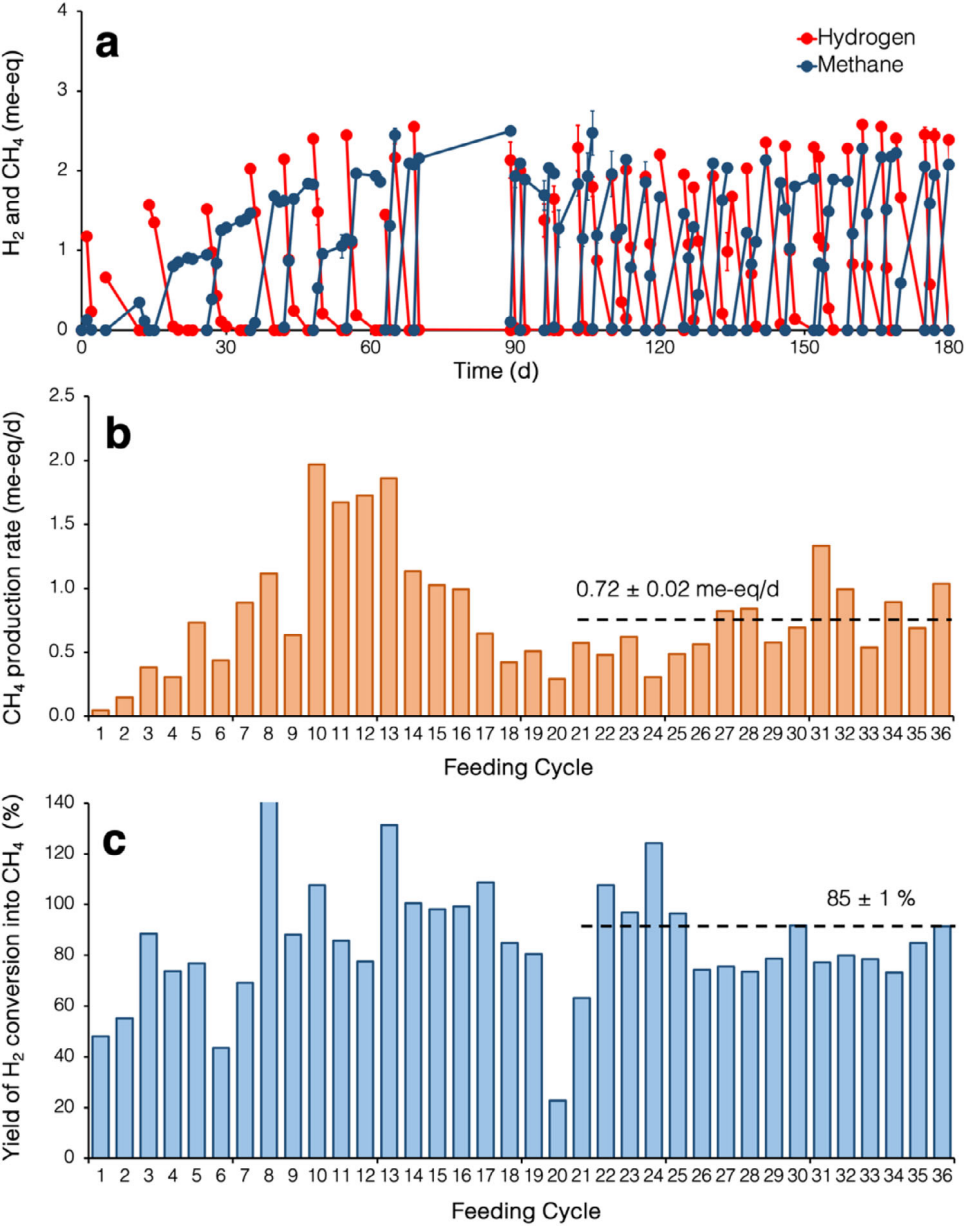
Kinetic characterization of the hydrogenotrophic methanogenic enrichment culture used for biomethanation tests. a) Time course of hydrogen consumption and methane generation over 36 successive feeding cycles. Error bars represent the standard error of replicated (*n* = 2) analyses. At the end of each cycle, the culture was flushed with nitrogen gas to remove unconsumed gaseous substrates, and a fixed volume of liquid phase was removed and replaced with fresh anaerobic medium to maintain an average hydraulic and cell retention time of ≈28 days. At the start of each cycle, the culture was supplied with H_2_ (50 mL) and HCO_3_^−^ (2.5 mL of a solution 10% wt/vol). b) Methane production rate during each feeding cycle. The dashed line reports the average value (± 1 standard error, *n* = 16) of the last 16 feeding cycles. c) Yield of hydrogen conversion into methane during each feeding cycle. The dashed line reports the average value (± 1 standard error, *n* = 16) of the last 16 feeding cycles.

The hydrogenotrophic methanogenic culture used as inoculum for the hereafter described (see paragraph 3.2) biomethanation batch experiments was originally enriched from an anaerobically digested sewage sludge, deriving from a municipal wastewater treatment plant. Prior to being used, the culture was maintained in a 0.5‐L fill‐and‐draw anaerobic bioreactor, regularly fed with H_2_ and CO_2_ as the sole electron donor and acceptor, respectively. Specifically, the bioreactor was operated for over 180 days, during which it collectively received 36 spikes of H_2_ (**Figure** **1**a). The average hydraulic and cell retention time was set at 28 days. The time between two successive H_2_ spikes was hereafter referred to as a “feeding cycle.” During the initial 20 cycles, the rate of methane production was unstable, clearly showing a peak value of ≈1.8 me‐eq/d in correspondence the cycles 10–13, prior to stabilizing at a lower level (Figure 1b). Conversely, during this initial period, the yield of H_2_ conversion into CH_4_ remained stable at 87 ± 7% (Figure 1c).

Upon reaching pseudo‐steady state conditions (i.e., approximately from the 21st feeding cycle, onward), methane was stably produced at an average rate of 0.72 ± 0.02 me‐eq/d (Figure 1b), with a corresponding yield of H_2_ conversion into CH_4_ of 85 ± 1% (Figure 1c).

At the end of the 36th feeding cycle, the archaeal and bacterial composition of the microbial community was analyzed. As expected, analyses revealed that the community was dominated by hydrogenotrophic methanogenic archaea, namely *Methanobrevibacter arboriphilus* and *Methanobacterium formicicum*, though it also contained H_2_‐consuming acetogenic bacteria, namely *Sporomusa* spp. and *Acetobacterium* spp., which accounted together for over 80% of total bacteria. Despite an anaerobically digested sludge was originally used as inoculum of the culture, the absence of acetoclastic methanogens is consistent with the long‐term enrichment using CO_2_ and H_2_ as the sole carbon and energy source.

### 2.2 Impact of Magnetite Nanoparticles Supplementation on the Kinetics of Biomethanation Reaction

At the end of the 36th feeding cycle, the enriched hydrogenotrophic methanogenic culture was used as inoculum for biomethanation batch experiments carried out in the presence of different concentrations of magnetite nanoparticles (i.e., 0, 1.25, and 2.5 gFe L^−1^). To this aim, magnetite nanoparticles synthesized accordingly to a previously published protocol,^[^ ^16^ ^]^ and having an average diameter of 3.8 ± 0.2 nm, were used. For each condition, triplicate microcosms were setup to ensure reproducibility.

**Figure 2:**
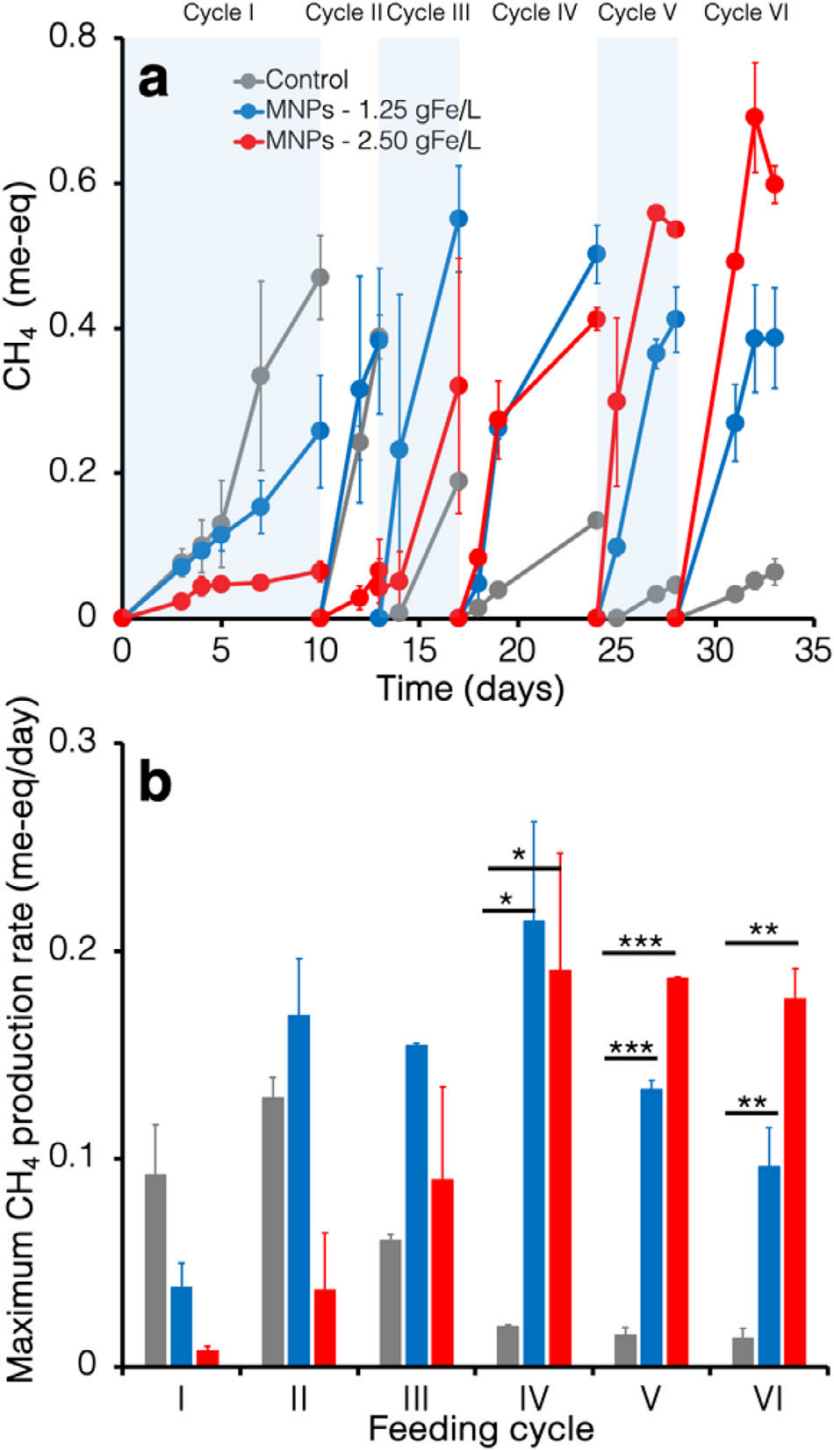
Effect of magnetite concentration on methanogenic activity. a) Time course of methane generation and B) maximum methane production rates in microcosms supplemented with increasing concentrations (0, 1.25, and 2.5 gFe L^−1^) of magnetite nanoparticles, over six successive feeding cycles. Error bars represent the standard error of replicates (for the control microcosm *n* = 3; for magnetite supplemented microcosms *n* = 2). Statistics, herein reported for the last three feeding cycles, were performed using two‐sided Student's *t* test (^*^ *p* value <0.05; ^**^ *p* value <0.01; ^***^ *p* value <0.001).

During the first feeding cycle methane production (and the corresponding hydrogen utilization, see Figure S1, Supporting Information) commenced with no lag phase in all microcosms (**Figure** **2**a), although the unamended controls displayed a higher maximum methane production rate (0.09 ± 0.02 me‐eq/d) compared to the microcosms supplemented with 1.25 gFe L^−1^ (0.04 ± 0.01 me‐eq/d) and 2.5 gFe L^−1^ (0.008 ± 0.002 me‐eq/d) of magnetite nanoparticles (Figure 2b). This finding possibly suggests that in the short‐term, magnetite nanoparticles exerted some form of stress on microbial activity.^[^ ^17^ ^]^ Interestingly, in the unamended controls, methanogenic activity peaked at the 2nd feeding cycles and then gradually declined prior to levelling off during the last three cycles (cycle 4–6). In microcosms supplemented with 1.25 and 2.5 gFe L^−1^ methanogenic activity increased over time, until stabilizing at values well above those observed in the unamended controls. As an example, in the 5th and 6th feeding cycle the maximum rate of methane production with 1.25 gFe L^−1^ of magnetite was 8.8‐ and 7‐fold higher than in the control, whereas in microcosms containing 2.5 gFe L^−1^ of magnetite it was 12‐ and 13‐fold higher. This finding clearly points to magnetite nanoparticles enhancing the kinetics of H_2_‐dependent biomethanation reaction. Clearly, longer‐term studies are needed to verify the stability and durability of the observed effect.

### 2.3 Analysis of the Microbial Composition of the Methanogenic Communities Supplemented with Magnetite Nanoparticles

**Figure 3:**
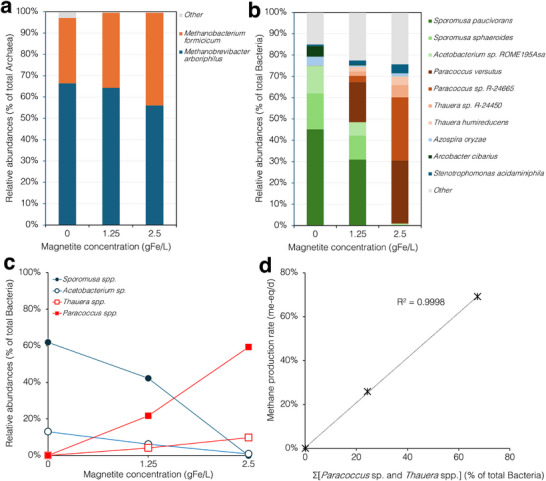
Effect of magnetite concentration on the composition of the methanogenic community. a) Relative abundance of archaeal and b) bacterial species in samples taken from the different microcosms at the end of the 6^th^ feeding cycle. c) Effect of magnetite nanoparticles concentration on the relative abundance of the H_2_‐oxidizining bacteria *Sporomusa paucivorans*, *Sporomusa sphaeroides*, *Acetobacterium* sp. ROME195Asa, *Paracoccus versutus*, *Paracoccus* sp. R‐24665, *Thauera humireducens* and *Thauera* sp. R‐24450. d) Linear correlation between the cumulative relative abundance of *Paracoccus* spp. and *Thauera* spp. and the rate of methane production in the microcosms supplemented with different amounts of magnetite nanoparticles.

At the end of the experiment, samples from the different microcosms were analyzed for identifying the main archaeal and bacterial components of the microbial communities. As far as archaea are concerned, analysis revealed that in the unamended controls, the community was dominated by hydrogenotrophic methanogens, namely *Methanobrevibacter arboriphilus* (accounting for 66.4% of total archaea) and *Methanobacterium formicicum* (30.6%). Interestingly, the relative abundance of these microorganisms was only marginally affected by the presence and concentration of magnetite nanoparticles (**Figure** **3**a).

Regarding bacteria, the unamended control revealed the presence of a rather diverse community in which H_2_‐oxidizing acetogens belonging the genus *Sporomusa*, namely *Sporomusa paucivorans* (accounting for 45.2% of total bacteria), *Sporomusa sphaeroides* (16.8%) and *Acetobacterium* sp. ROME195Asa (13.0%), accounted collectively for over 75% of total bacteria (Figure 3b). Notably, in magnetite supplemented microcosms, the relative abundance of acetogens decreased in a dose‐dependent manner (i.e., ≈48.5% with 1.25 gFe L^−1^ and 0.9% with 2.5 gFe L^−1^ of magnetite). This decrease was mirrored by a nearly corresponding increase in the abundance of other H_2_‐oxidazing bacteria, namely *Paracoccus* spp. (i.e., *P. versutus* and *P*. sp. R‐24665) and to a lesser extent *Thauera* spp., which collectively accounted for <0.1% of total bacteria in the unamended control, 25.8% with 1.25 gFe L^−1^ of magnetite and 69.1% with 2.5 gFe L^−1^ of magnetite (Figure 3c).

Apparently, the relative abundance of *Paracoccus* sp. and *Thauera* spp. was linearly correlated with the observed rate of methane production by the microbial culture, though these microorganisms are clearly not directly involved in methane generation, lacking the required enzymatic machinery (Figure 3d). Interestingly, *Paracoccus* and *Thauera*, though phylogenically distant microorganisms (belonging to the family of Rhodobacteraceae and Zoogloeaceae, respectively), share numerous similar metabolic traits, particularly in their roles as denitrifiers in various environments.^[^ ^18^, ^19^ ^]^ Indeed, they are both capable of chemolitotrophic growth using H_2_ as electron donor and nitrate or oxygen as respiratory electron acceptors. Clearly, due to the lack of their physiological electron acceptor (e.g., NO_3_^−^) in the reaction media, their almost complete absence in the enrichment culture (i.e., the inoculum), as well as in the unamended control microcosms, is an expected finding. On the other hand, their noticeable enrichment in the magnetite‐supplemented microcosms (up to nearly 70% of total bacteria) at the expense of the H_2_‐oxidizing acetogens indicates that these latter were likely out‐competed for the consumption of H_2_. This is consistent with the reported faster growth kinetics on hydrogen of *Paracoccus* spp. and *Thauera* spp. relative to their acetogenic counterpart (i.e., *Sporomusa* spp. and *Acetobacterium* spp.).^[^ ^20^, ^21^, ^22^ ^]^

Since during each feeding cycle, the yield of H_2_ conversion into CH_4_ in the magnetite‐supplemented microcosms remained extremely high (i.e., ranging between 78% and 107%), it is apparent that *Paracoccus* and *Thauera* species somehow participated to the overall biomethanation process, possibly being involved in the initial H_2_ oxidation and the subsequent interspecies extracellular electron transfer to the methanogens, with such process being observed only in the presence of magnetite nanoparticles.

Notably, the extracellular electron transfer capabilities of *Paracoccus* spp., most likely triggered by the numerous different redox‐active proteins present in its respiratory chain which span a broad range of redox potentials,^[^ ^23^ ^]^ have been previously documented in the literature. As an example, a previous study^[^ ^24^ ^]^ examined the microbial community of an MFC fed with formic acid for more than 1 year and determined using 16S rRNA gene cloning and fluorescent in situ hybridization that members of the *Paracoccus* genus comprised most (≈30%) of the anode community. A *Paracoccus* isolate obtained from this biofilm produced only 5.6 mW m^−2^, whereas the original mixed culture produced up to 10 mW m^−2^. In another study, a *Paracoccus* strain isolated from rumen fluid demonstrated electrochemical activity and successfully generated power in an MFC.^[^ ^25^ ^]^

### 2.4 Analysis of Soluble Iron Species: Ruling Out the Possibility of Mediated Interspecies Electron Transfer Between *Paracoccus* spp. and Methanogens

At the end of the microcosm experiments, filtered (0.2‐µm) liquid samples from the different microcosms were analyzed to determine the concentration of soluble iron species (Fe^2+^ and Fe^3+^), possibly deriving from the supplemented magnetite nanoparticles and/or from their biologically‐catalyzed transformation. The dissolved total Fe concentration (i.e., the sum of Fe^2+^ and Fe^3+^) was 4.1 ± 0.2 µmol L^−1^ in the unamended control and 2.5 ± 0.1 and 2.1 ± 0.1 µmol L^−1^ in the microcosms supplemented with 1.25 and 2.5 gFe L^−1^ of magnetite nanoparticles, respectively. This finding confirms the chemical and biological stability of the synthesized magnetite nanoparticles and allows to rule out a possible contribution of soluble iron species on the observed enhancement of methane production, both as stimulatory nutrients and/or as redox shuttles facilitating IET among *Paracoccus* spp. and methanogens, as previously reported.^[^ ^26^ ^]^

### 2.5 Microscopy Analyses of Magnetite Supplemented Microcosms

**Figure 4:**
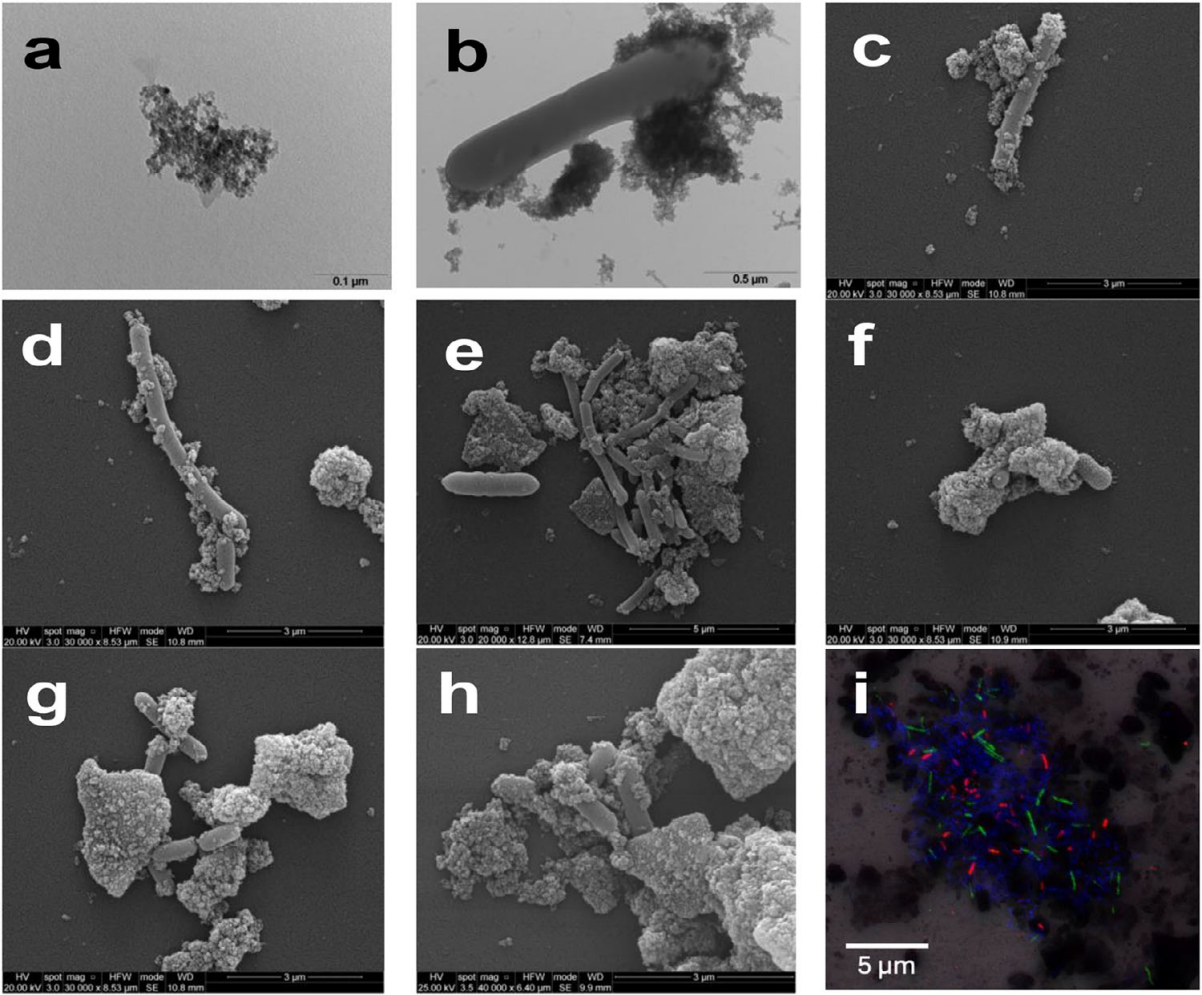
Visualization of samples taken from the different microcosms using different microscopy techniques. a) TEM negative staining of magnetite aggregates in which the elemental nanoparticles have an average size of 3.9 ± 0.7 nm. b) TEM and c) SEM micrographs of a bacillus in tight contact with magnetite nanoparticle aggregates. d,e) SEM micrographs showing compact aggregates of microbial cells interconnected by a network of magnetite nanoparticles. f–h) SEM micrographs of aggregates of nanoparticles encasing microbial cells. i) CLSM combined images showing the spatial distribution of archaea (green) and Alphaproteobacteria (red) cells identified by FISH in aggregates from the magnetite‐supplemented cultures. DAPI‐stained cells appear blue. Images b, c, d, f, g, and h refer to samples taken from the microcosms supplemented with 2.5 gFe L^−1^ of magnetite, images E and I refer to samples taken from the microcosms supplemented with 1.25 gFe L^−1^ of magnetite.

To verify the existence of specific interactions among the supplemented magnetite nanoparticles and members of the microbial communities, a suite of microscopy analyses was applied on samples taken from the different microcosms. **Figure** **4**a shows a TEM negative staining of the synthesized magnetite nanoparticles in which individual particles appeared nearly spherical, with an average diameter of ≈4 nm, in agreement with the shape and dimensions reported in a previous study where the same synthesis protocol was applied.^[^ ^16^ ^]^ Interestingly, both TEM and SEM, revealed that nanoparticles attached firmly to the outer membrane of microorganisms (Figure 4b,c), possibly indicating the establishment of specific chemical or electrostatic interactions with components of the cells’ membranes.

The presence of magnetite also markedly affected the microbial communities at ultrastructural level. In magnetite supplemented samples cells tended to form compact aggregates in which cells were apparently kept together by a structured network of magnetite nanoparticles (Figure 4d,e). Energy dispersive X‐ray spectroscopy (EDS) analyses of this network confirmed the abundance of iron and oxygen, in agreement with the elemental composition of magnetite (Figure S2, Supporting Information). The size of aggregates, in magnetite supplemented samples, could vary considerably, spanning from a few micrometers (and comprising only few cells) up to ≈30–40 µm. By contrast, in the unamended controls, larger aggregates of different morphologies, surrounded by an extracellular polymeric matrix were typically observed (Figure S2, Supporting Information). At high magnifications, in the samples with the highest applied concentration of magnetite, some cells appeared to be almost entirely covered by layers of magnetite nanoparticles and to form very compact clusters (Figure 4f). In some cases, microbial cells could hardly be recognized on the surface of these clusters, being almost entirely embedded within the conductive network of nanoparticles (Figure 4g,h). Visualization of the samples with confocal laser scanning microscopy (CLSM), in combination with fluorescent in situ hybridization (FISH) targeting methanogens (using the oligonucleotide probe specific for *Archaea*) and *Paracoccus* spp. (using an oligonucleotide probe specific for *Alphaproteobacteria*) revealed the co‐existence of these microorganisms within the magnetite aggregates, thus providing a further line of evidence of the existence of a complex interspecies interaction (Figure 4i).

### 2.6 Occurrence of a Possible Mutualistic Cooperation Between *Paracoccus*, *Thauera*, and Methanogens Based on IET

The most straightforward effect of magnetite supplementation to the microbial community is the noticeable enhancement (up to 13‐fold) in the kinetics of the biomethanation reaction. Interestingly, this enhancement was mirrored by a remarkable enrichment of *Paracoccus* spp. (and to a lesser extent of *Thauera* spp.) and a corresponding reduction in the abundance of H_2_‐oxidizing acetogens (e.g., *Sporomusa* spp. and *Acetobacterium* spp.). This finding suggests that *Paracoccus* spp. outcompeted acetogens for H_2_ consumption thanks to their higher growth rates.^[^ ^22^ ^]^ On the other hand, the lack in the reaction medium of the physiological electron acceptor (i.e., NO_3_^−^) typically used by *Paracoccus* spp. (as well as *Thauera* spp.), raises into question the employed mechanisms of energy generation used by these microorganisms for growth. One possibility is that metabolic electrons deriving from H_2_ oxidation were ultimately discharged to a membrane‐bound redox site within a partner methanogenic organism, virtually serving as an alternative electron acceptor. Possibly, this type of opportunistic behavior enabled *Paracoccus* strains to proliferate and, thus, it was most likely an energy‐yielding process.

So far, the capability for extracellular electron uptake of methanogens has been mostly observed in acetoclastic methanogens such a *Methanosarcina* and *Methanothrix* species.^[^ ^27^ ^]^ The only hydrogenotrophicmethanogen reported so far to grow via DIET in syntrophic association with *Geobacter metallireducens*, is *Methanobacterium* strain YSL,^[^ ^28^ ^]^ although the underlaying mechanisms of electron uptake by this microorganism remain unknown. Interestingly, recent studies revealed that the archaellum (i.e., an extracellular protein filament) of the hydrogenotrophicmethanogen *Methanospirillum hungatei*, is conductive, thus pointing to its possible role as electron conduit during extracellular electron uptake.^[^ ^29^ ^]^

**Figure 5:**
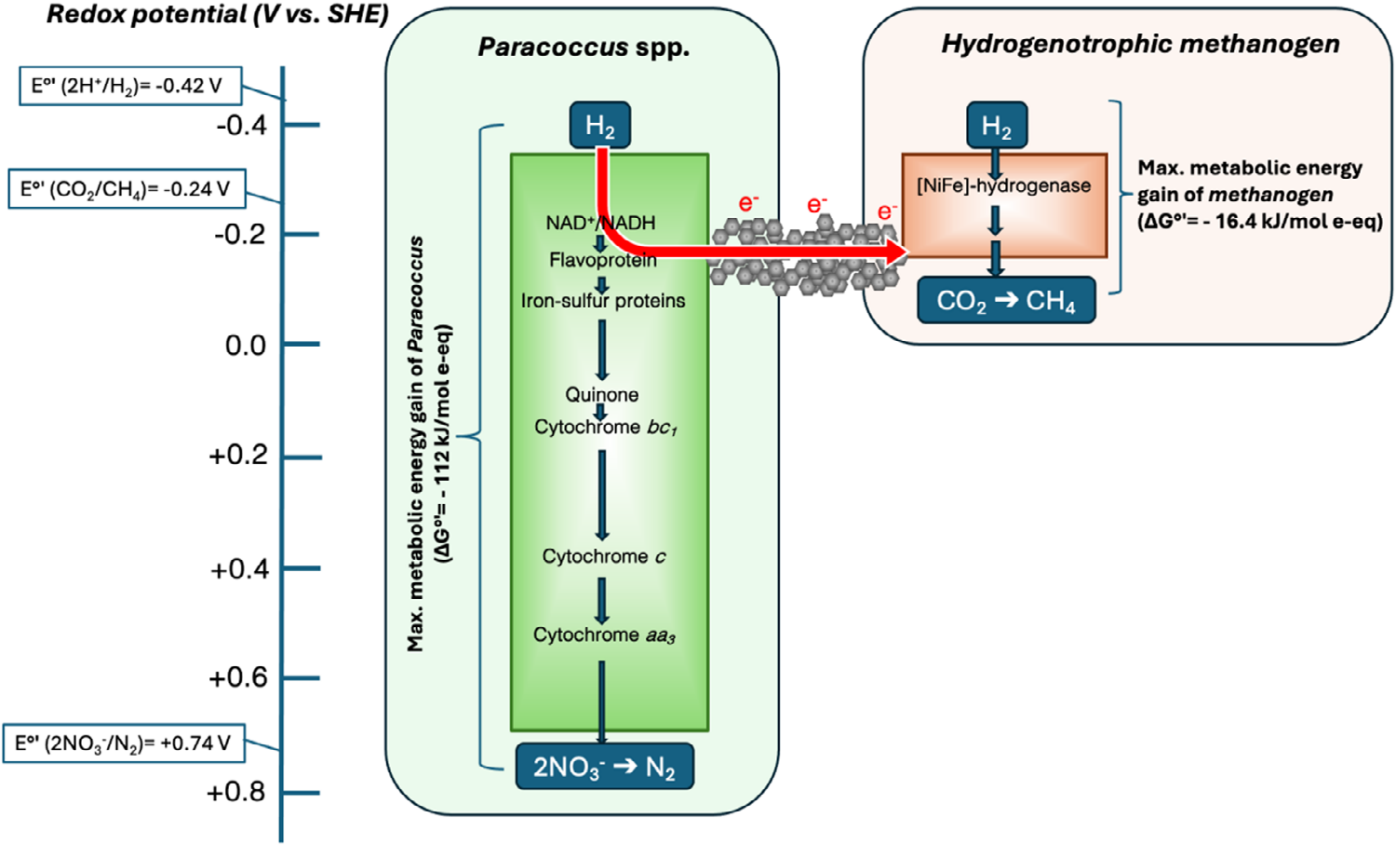
Proposed model depicting electron flows in *Paracoccus* sp. and the hydrogenotrophic methanogens with or without DIET‐based mutualism. The red arrow indicates the flow of electrons from the primary electron donor (i.e., H_2_) to the terminal electron acceptor (i.e., CO_2_) during the DIET‐based mutualistic cooperation between the microorganisms. This shortcut was enabled by the magnetite nanoparticles and occurred in the absence of nitrate.

Regardless the involved electron uptake route and mechanism, in the present study, methanogens clearly benefited from the higher electron flux fueled by *Paracoccus* and *Thauera* and “travelling” along the magnetite nanoparticles, which enabled a substantially higher methane production, compared to what they could produce alone. This, however, came at the expenses of a necessarily lower energy availability for the methanogens. Indeed, in the absence of such a mutualistic cooperation, the methanogens could potentially exploit all the energy deriving from hydrogen oxidation coupled to carbon dioxide reduction to methane (i.e., 16.4 kJ mol^−1^ e‐eq) (**Figure** **5**).^[^ ^30^ ^]^ Now, the same amount of metabolic energy had to be shared among (at least) the two participating partners, according to the hypothesized model depicted in Figure 5. Clearly, further investigations, potentially involving pure culture studies, are warranted to validate the proposed model, elucidate the biochemical “machinery” involved, and quantify both the energy generation and the growth yields of the individual species participating in this novel cooperative metabolism.

Over the past decades, the respiratory chain of *Paracoccus species* and particularly of *P. denitrificans* has been the subject of extraordinarily intense investigations.^[^ ^31^, ^32^ ^]^ The remarkable similarities among the electron transfer chain and oxidative phosphorylation system in *Paracoccus* with the mitochondrion of Eukaryota have raised the fascinating hypothesis that this latter organelle may have evolved from the integration of an endosymbiotic Alphaproteobacterium like *Paracoccus* into the host cell of a newly discovered group of archaea, known as the Asgards archaea.^[^ ^33^, ^34^ ^]^ Although controversy persists as this symbiotic relationship between *Paracoccus* and archaea has originated the first eukaryotic cell,^[^ ^35^ ^]^ it is unquestionable that a number of similarities exist with the mutualistic metabolism, triggered by magnetite nanoparticles, observed in the present study in which *Paracoccus* virtually served as a sort of “free‐living” mitochondrion of the methanogenic Archaea.^[^ ^36^ ^]^

## 3 Conclusion

The results of this study clearly demonstrate that magnetite supplementation is an effective strategy to enhance the kinetics of CH_4_ generation from H_2_ and CO_2_ in a mixed microbial culture, with promising implications for the advancement of power‐to‐methane technologies, such as biogas upgrading. The observed dose‐dependent enrichment of *Paracoccus* spp. and *Thauera* spp. in magnetite‐amended microcosms points to the establishment of a mutualistic cooperation with methanogens, likely sustained by IET. Beyond its practical relevance, this work clearly broadens the current understanding of interspecies electron transfer (IET) in anaerobic microbial communities, emphasizing its role beyond classical syntrophic interactions. On a broader perspective, herein reported results raise the intriguing hypothesis that the H_2_‐oxidizing metabolic potential of *Paracoccus* spp. could be harnessed to enhance other IET‐driven hydrogenation processes of environmental or industrial interest. Future studies should also investigate the environmental occurrence and ecological role of *Paracoccus* spp. in magnetite‐rich subsurface environments, where it may act as an overlooked, indirect contributor to methane emissions.

## 4 Experimental Section

### Enrichment of the Hydrogenotrophic Methanogenic Culture in an Anaerobic Bioreactor

The hydrogenotrophic methanogenic culture used as inoculum for the hereafter described microcosm experiments was maintained for ≈180 days in a 0.5 L anaerobic bioreactor, with a working volume of 0.4 L. The bioreactor consisted of a glass bottle, sealed by rubber stoppers, screw caps, and mixed continuously at 150 rpm in an orbital shaker, maintained at 22 ± 1 °C. The bioreactor was initially seeded with 20 mL of an anaerobically digested municipal sludge (Rome, Italy WWTP), that was diluted to a final volume of 400 mL with anaerobic mineral medium containing the following substances (in g L^−1^): NaHCO_3_ 4.2; NH_4_Cl 0.5; MgCl_2_•6H_2_O 0.1; CaCl_2_•2H_2_O 0.05; K_2_HPO_4_ 8; KH_2_PO_4_ 8. To the mineral medium, 10 mL L^−1^ of trace metal solution and 1 mL L^−1^ of vitamin solution were added. The trace metal solution^[^ ^37^ ^]^ contained (in g L^−1^): Nitrilotriacetic acid 4.5; FeSO_4_•7H_2_O 0.556; MnSO_4_•H_2_O 0.086; CoCl_2_•6H_2_O 0.17; ZnSO_4_•7H_2_O 0.21; H_3_BO_3_ 0.019; NiCl_2_ 0.02; Na_2_MoO_4_ 0.01. The vitamin solution^[^ ^38^ ^]^ was composed of (in g L^−1^): Biotin (B_7_) 0.02; Folic acid (B_9_) 0.02; Pyridoxine (B_6_) 0.1; Thiamine (B_1_) 0.05; Riboflavin (B_2_) 0.05; Nicotinic acid (B_3_) 0.05; Pantothenic acid (B_5_) 0.05; Cyanocobalamin (B_12_) 0.002; 4‐aminobenzoic acid (B_10_) 0.05. The final pH of the medium was ≈7.0. Throughout the study, the bioreactor was operated in a fill‐and‐draw mode. In brief, nearly on a weekly basis, a fixed volume of liquid phase was removed and replaced with fresh anaerobic medium; subsequently the headspace was flushed with nitrogen gas to remove the produced methane. Finally, a dose of hydrogen (50 mL) and bicarbonate (2.5 mL from a 10% wt vol^−1^ solution) were added. The average hydraulic and cell retention time was 28 days.

### Setup of the Microcosm Study

Microcosms were setup in 120‐mL, anaerobic serum bottles (40‐mL working volume) that were sealed with Teflon‐faced butyl rubber stoppers and aluminum crimps. Three different treatments were setup. Unamended controls (in triplicate) contained 15 mL of the methanogenic culture (as inoculum), 21 mL of mineral medium, and 4 mL of bicarbonate (10% wt vol^−1^); microcosms supplemented with a lower concentration of magnetite nanoparticles (1.25 gFe L^−1^) contained 15 mL of the methanogenic culture (as inoculum), 16 mL of mineral medium, 5 mL of a suspension of magnetite nanoparticles, and 4 mL of bicarbonate (10% wt vol^−1^); finally microcosms supplemented with a higher concentration of magnetite nanoparticles (2.50 gFe L^−1^), contained 15 mL of the methanogenic culture (as inoculum), 11 mL of mineral medium, 10 mL of a suspension of magnetite nanoparticles, and 4 mL of bicarbonate (10% wt vol^−1^). Magnetite‐supplemented microcosms were setup in duplicate. The suspension of magnetite nanoparticles was prepared as reported elsewhere,^[^ ^12^ ^]^ following a previously published protocol.^[^ ^16^ ^]^ Upon preparation, all the microcosms were flushed with nitrogen gas to ensure strictly anaerobic conditions and were spiked with hydrogen (20 mL). Twice a week a 2‐mL liquid sample was removed from each microcosm, for pH measurements, and replaced with anaerobic mineral medium. Hydrogen was re‐added every time analyses indicated it was almost completely consumed. During the incubation, all the microcosms were kept at 22 ± 1 °C in an orbital shaker (150 rpm).

### Analyses

The concentration of hydrogen and methane was measured by injecting 50 µL of gaseous samples with a gastight syringe (Hamilton, Reno, NV, USA) into a gas‐chromatograph equipped with a thermal conductivity detector (TCD, Agilent 8860, GC system, Santa Clara, CA, USA). Gas quantification was performed using external standards. All chemicals were of the highest grade available and were purchased from Sigma–Aldrich now Merck (St. Louis, MO, USA), unless otherwise stated. Total Fe, Fe(II), and Fe(III) were determined spectrophotometrically.^[^ ^39^ ^]^ To compute mass and electron balances, the molar amounts of hydrogen and methane were converted in electron equivalents (e‐eq) using the following conversion factors: 2 e‐eq mol^−1^ for hydrogen and 8 e‐eq mol^−1^ for methane.

### DNA Extraction, Library Preparation, and Long‐Read Sequencing of Microbial Cultures

Samples (50 mL) for microbial characterization were collected from the enrichment culture and the microcosms using a sterile syringe The samples were then filtered through hydrophilic polycarbonate membranes (0.2 µm pore size, 25 mm diameter, Millipore). The filters were immediately used for genomic DNA extraction, which was carried out using the DNeasy PowerLyzer PowerSoil Kit (QIAGEN) according to the manufacturer's instructions.

Amplicon libraries for the archaea 16S rRNA gene variable regions 4–9 (aV49‐A) were prepared using a custom protocol. Up to 25 ng of extracted DNA was used as template for PCR amplification, and each PCR reaction (50 µL) contained 0.2 mm dNTP mix, 0.01 units of Platinum SuperFi DNA Polymerase (Thermo Fisher Scientific, USA), and 500 nm of each forward and reverse primer in the supplied SuperFI Buffer. PCR was done with the following program: Initial denaturation at 98 °C for 3 min, 25 cycles of amplification (98 °C for 30 s, 62 °C for 20 s, 72 °C for 2 min) and a final elongation at 72 °C for 5 min. The forward and reverse primers used include custom 24 nt barcode sequences followed by the sequences targeting aV49‐A: [515FB] GTGYCAGCMGCCGCGGTAA and [SSU1000ArR] GGCCATGCAMYWCCTCTC.^[^ ^40^, ^41^, ^42^, ^43^ ^]^ The resulting amplicon libraries were purified using the standard protocol for CleanNGS SPRI beads (CleanNA, NL) with a bead to sample ratio of 3:5. DNA was eluted in 25 µL of nuclease free water (Qiagen, Germany). Sequencing libraries were prepared from the purified amplicon libraries using the SQK‐LSK114 kit (Oxford Nanopore Technologies, UK) according to manufacturer protocol with the following modifications: 500 ng total DNA was used as input, and CleanNGS SPRI beads for library clean‐up steps. DNA concentration was measured using Qubit dDNA HS Assay kit (Thermo Fisher Scientific, USA). Gel electrophoresis using Tapestation 2200 and D1000/High sensitivity D1000 screentapes (Agilent, USA) was used to validate product size and purity of a subset of amplicon libraries. The resulting sequencing library was loaded onto a PromethION R10.4.1 flowcell and sequenced using the MinKNOW 24.02.10 software (Oxford Nanopore Technologies, UK). Amplicon libraries for the bacterial 16S rRNA gene were prepared using the Oxford Nanopore 16S Barcoding Kit (SQK‐16S024), according to the manufacturer's instructions. The genomic library was loaded onto a FLO‐MIN106D (chemistry R9.4.1) flow cell and sequenced using the MinION Mk1B device (Oxford Nanopore Technology, ONT). All DNA sequencing reads were deposited in the Sequence Read Archive (SRA) under Bioproject number PRJNA1132306.

### Bioinformatics

Reads were basecalled and demultiplexed with MinKNOW guppy Dorado 7.3.9 using the super accurate basecalling algorithm (config dna_r10.4.1_400bps_sup.cfg and dna_r9.4.1_450bps_sup.cfg) and custom barcodes for archaea. Bacteria and archaea 16S rRNA reads were filtered by length and quality score with Chopper v0.8.0 (https://github.com/wdecoster/chopper). For bacteria long reads, a minimum quality score of 9 was set, with lengths between 1400 and 1700 bp. For archaea, a minimum quality score of 20 was applied, with a minimum length above 500 bp. Taxonomic assignment and calculations of taxa abundances for the 16S rRNA reads of both archaea and bacteria were performed using EMU v3.4.5 (10.1038/s41592‐022‐01520‐4) against the Silva NR99 database v138.2. The EMU abundance data of each taxon are provided in the Supplementary material (Table S1, Supporting Information).

### Scanning and Transmission Electron Microscopy

Liquid samples taken from the different microcosms were left to adhere to poly‐lysine treated glass coverslips for 30 min at room temperature prior to being processed as described elsewhere.^[^ ^44^ ^]^ In brief, samples were fixed with glutaraldehyde 2.5% in a Na‐cacodilate buffer 0.1 m overnight at 4 °C, post‐fixed in osmium tetroxide 1% in the same buffer for 1 h at room temperature and dehydrated through graded series of ethanol solutions (from 30% to 100%). Then, ethanol was gradually substituted by hexamethyldisilazane (HMDS) through a 1:1 (ethanol: HMDS) incubation for 30 min, followed by pure HMDS for 30 min and then by a final drying process, totally removing HMDS and leaving it to evaporate under a chemical hood for 2 h. All dried coverslips were mounted on stubs, gold‐sputtered and analyzed by a Field Emission‐SEM (FE‐SEM) Quanta Inspect F (FEI – Thermo‐Fisher Scientific; Eindhoven – The Netherlands) equipped with an EDAX detector used for energy dispersive X‐ray spectroscopy (EDS) analysis on the microbial agglomerates. Representative spectra of three different points in three different agglomerates for each microcosm were produced at 30 kV.

Negative staining in Transmission Electron Microscopy (TEM) was performed as described previously.^[^ ^44^ ^]^ Briefly, 5 µL of the sample was adsorbed on copper carbon coated grids, air dried and stained by ammonium molybdate 4% pH 6.4 (5 µL). Then, the sample was air dried and observed by a PHILIPS EM208S TEM (FEI – Thermo Fisher Scientific; Eindhoven – The Netherlands) at 100 kV, equipped with the Megaview SIS camera II (Olympus).

High magnification TEM images of synthesized magnetite nanoparticles were analyzed by Fiji, Image J open source.^[^ ^45^ ^]^ Only peripherical particles were considered for collecting manual measures from the different agglomerates (N = 30).

### Fluorescent In Situ Hybridization and Confocal Laser Scanning Microscopy

Fluorescence in situ hybridization (FISH) analysis was performed on paraformaldehyde‐fixed samples according to a procedure described elsewhere.^[^ ^12^, ^46^ ^]^ Oligonucleotide probes specific for Alphaproteobacteria (ALF1b and ALF968 probes) and archaea (ARC915 probe) domains were used. Details of the employed oligonucleotide probes are available at probeBase.^[^ ^47^ ^]^ The hybridization was carried out in combination with DAPI staining to estimate total cell abundance. In order to visualize specific cells within the aggregates, FISH was combined with confocal laser scanning microscopy (CSLM; Olympus FV1000).^[^ ^48^, ^49^ ^]^ Archaea were excited with the 488 nm line of an Ar laser (excitation) and observed in the green channel from 500 to 530 nm (emission). Alphaproteobacteria were excited with the 543 nm line of a He–Ne laser and observed in the red channel from 550 to 660 nm.

### Statistical Analysis

All average data are presented as mean values ± standard error (SE). Statistical significance of differences between groups was assessed using a two‐sided Student's *t*‐test. Mean values were considered statistically different when *p* < 0.05. All statistical analyses were performed using GraphPad Prism software.

## Conflict of Interest

The authors declare no conflict of interest.

## Author Contributions

M.T. contributed to the conceptualization, formal analysis, writing – original draft, and visualization. J.I.S. contributed to the investigation, formal analysis, and visualization. C.C.V. contributed to the conceptualization, formal analysis, and writing – original draft. L.B. contributed to the investigation, formal analysis, visualization, and writing – original draft. F.I. contributed to the investigation, formal analysis, visualization, and writing – original draft. E.D. contributed to the investigation, formal analysis, visualization, and writing – original draft. D.U. contributed to the formal analysis and writing – original draft. B.M. contributed to the investigation, formal analysis, visualization, and writing – original draft. A.F. contributed to the formal analysis, visualization, and writing – original draft. A.P. contributed to the investigation, formal analysis, and visualization. S.F. contributed to the investigation, formal analysis, visualization, and writing – original draft. F.A. contributed to the conceptualization, formal analysis, visualization, writing – original draft, supervision, and funding acquisition. All authors edited the manuscript and approved the final version.

## Supporting information



Supporting Information

## Data Availability

The data that support the findings of this study are available from the corresponding author upon reasonable request.
