# Supplementary material for: Magnetite Nanoparticles Enhancing H2‐Driven Biomethanation in a Mixed Microbial Community
Source: Glob Chall. 2025 Aug 19;9(9):e00367. doi: 10.1002/gch2.202500367 (PMC12418360; doi:10.1002/gch2.202500367)
Supplement: Supplementary file 1 — Supporting Information [file GCH2-9-e00367-s001.docx]

Supporting Information

Magnetite Nanoparticles Enhancing H_2_-Driven Biomethanation in a Mixed Microbial Community

*Matteo Tucci,^[a]^ Jasper I Sabangan,^[a]^ Carolina Cruz Viggi,^[a]^ Lucia Bertaccini,^[b]^ Francesca Iosi,^[b]^ Emilio D’Ugo,^[c]^ Daniela Uccelletti,^[d]^ Bruna Matturro,^[a]^ Andrea Firrincieli,^[e]^ Agnese Piacentini,^[a,d]^ Stefano Fazi,^[a]^ and Federico Aulenta*^[a]^*


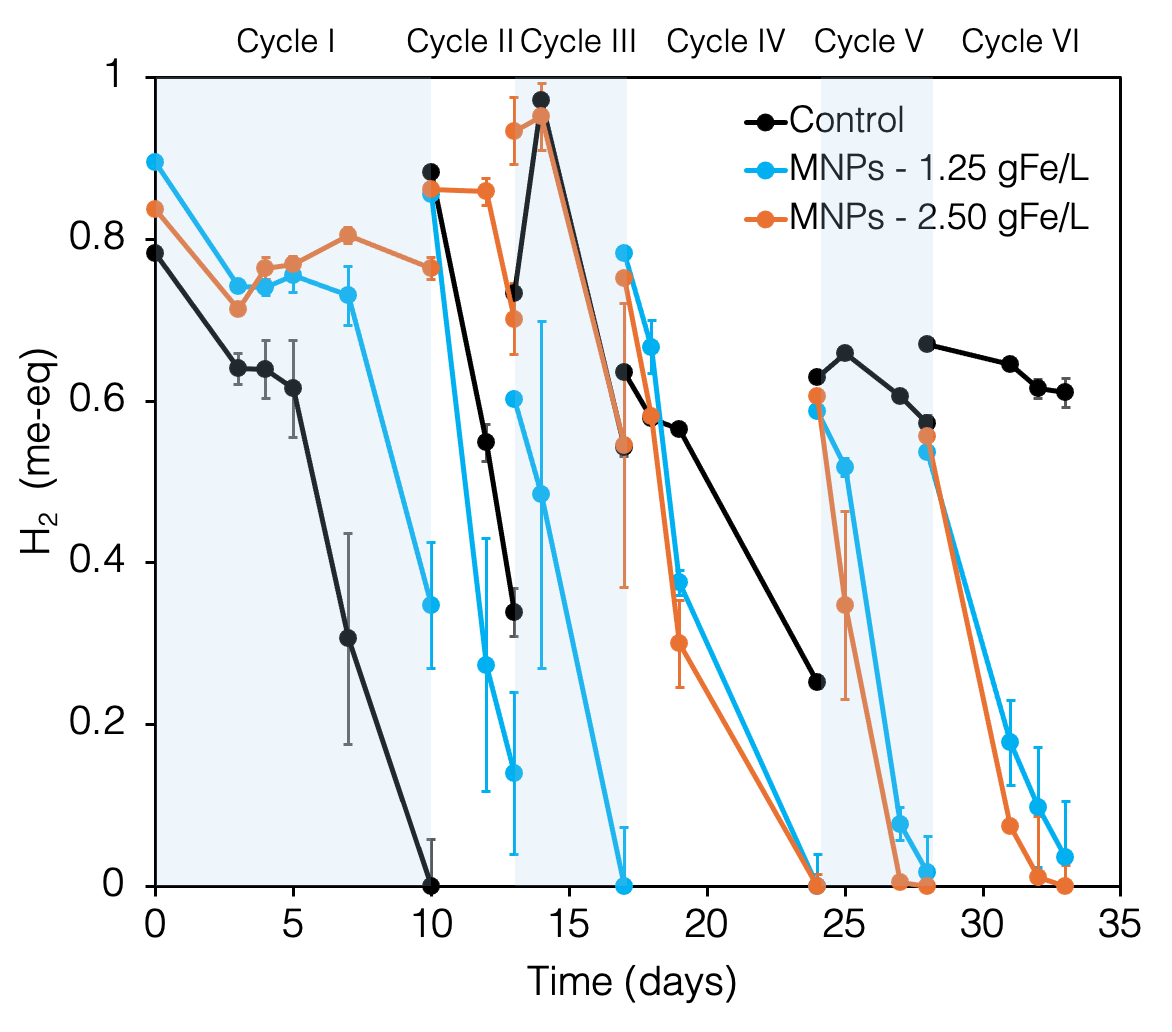


Figure S1. Effect of magnetite concentration on the time course of hydrogen utilization during the microcosm experiments shown in Figure 2.


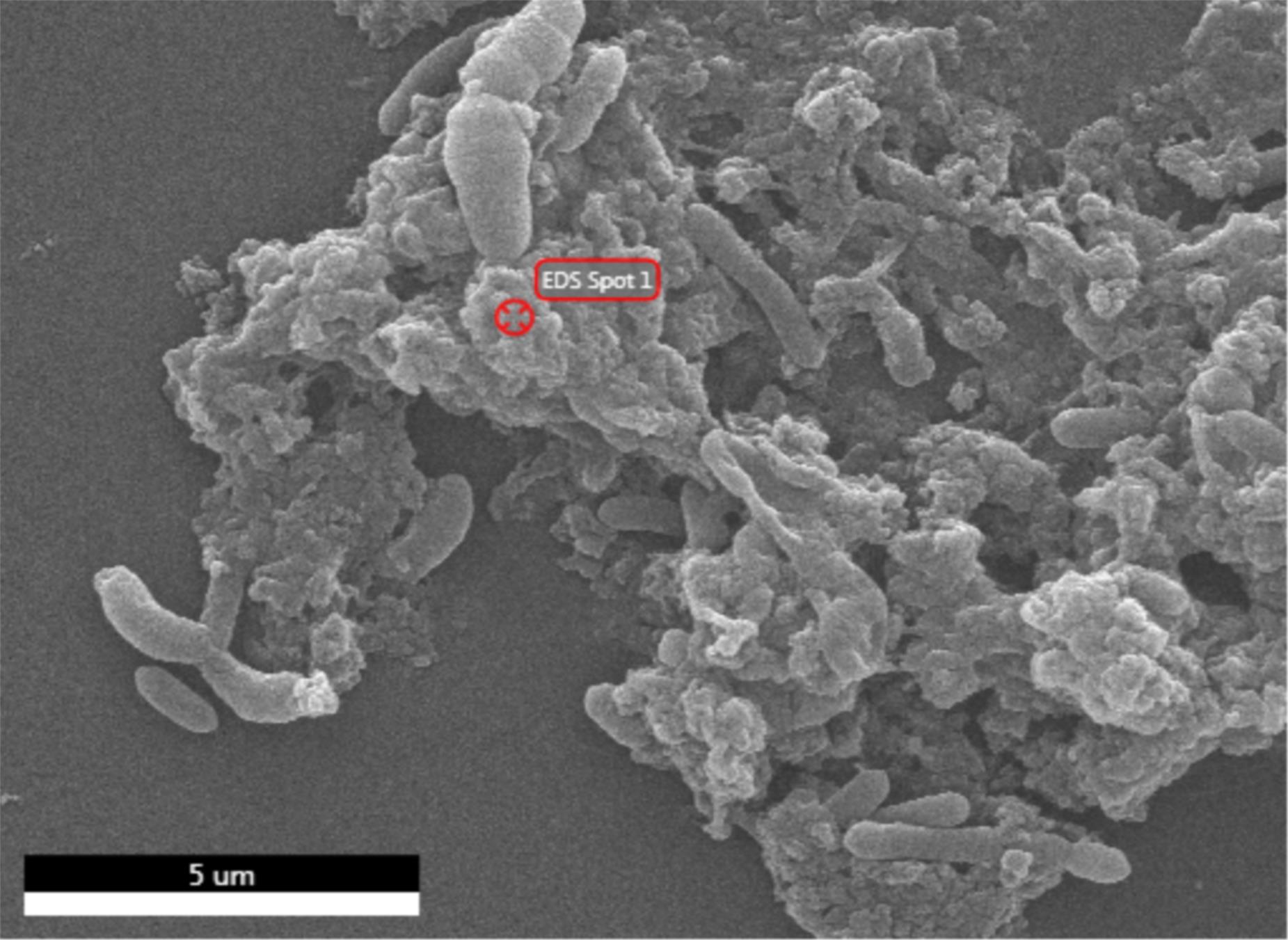

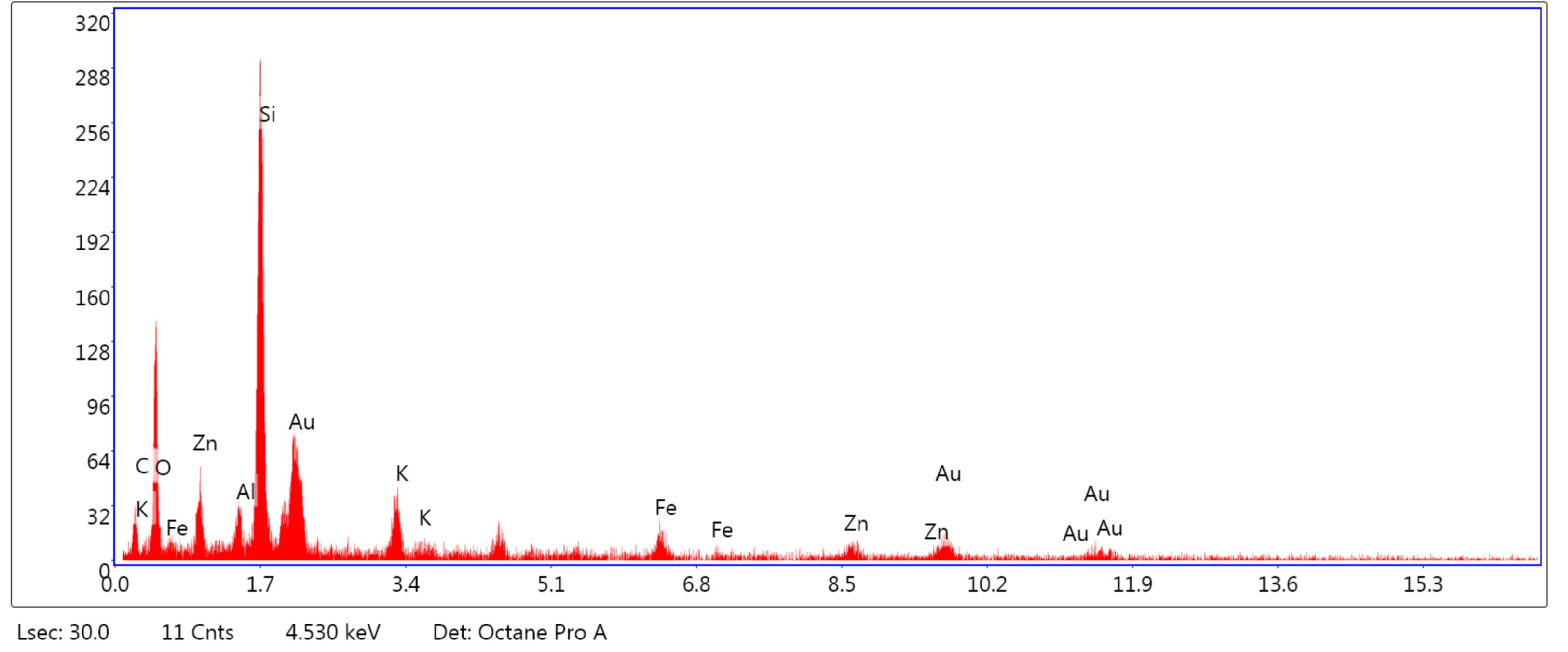


0 gFe/L


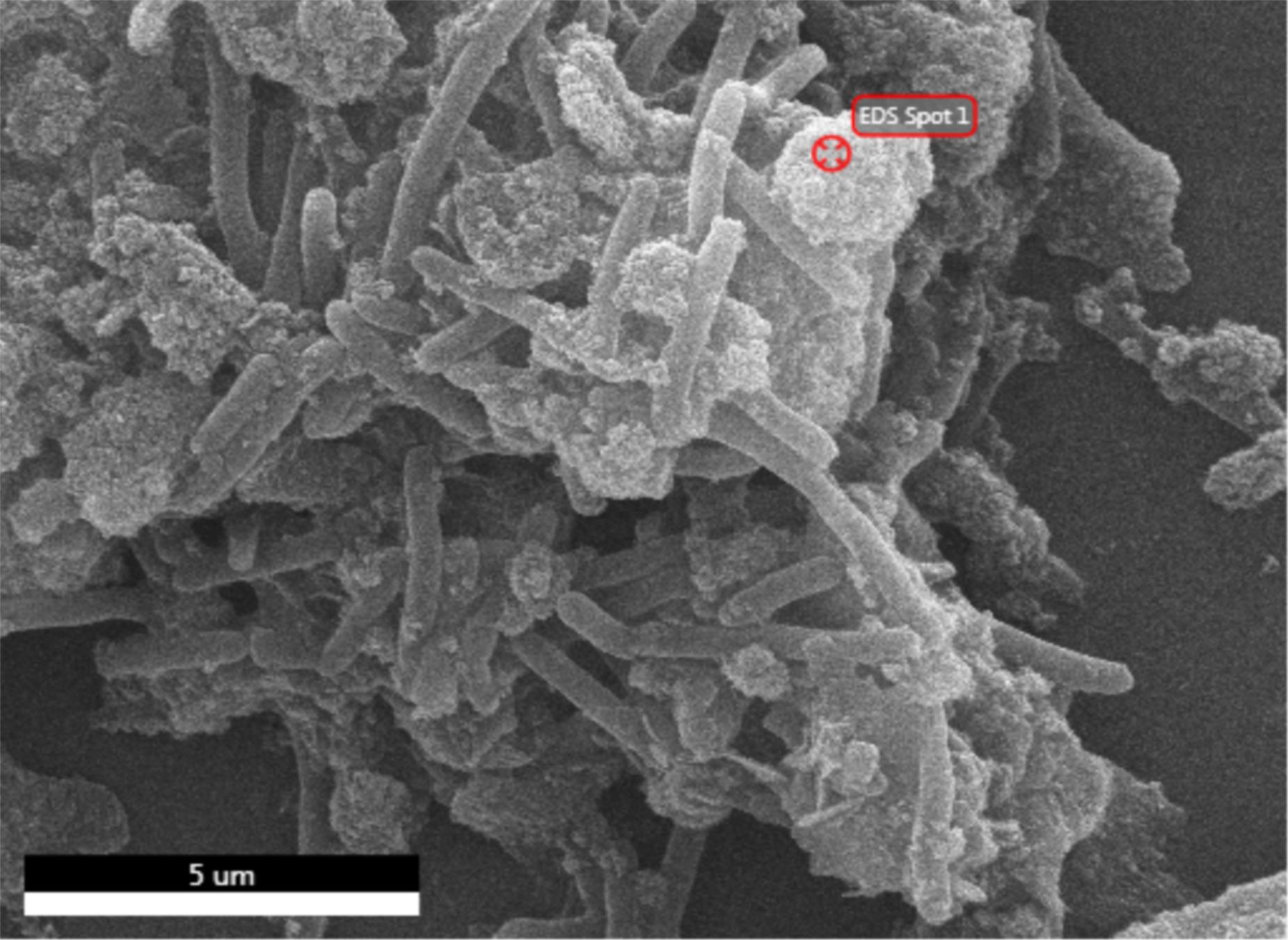

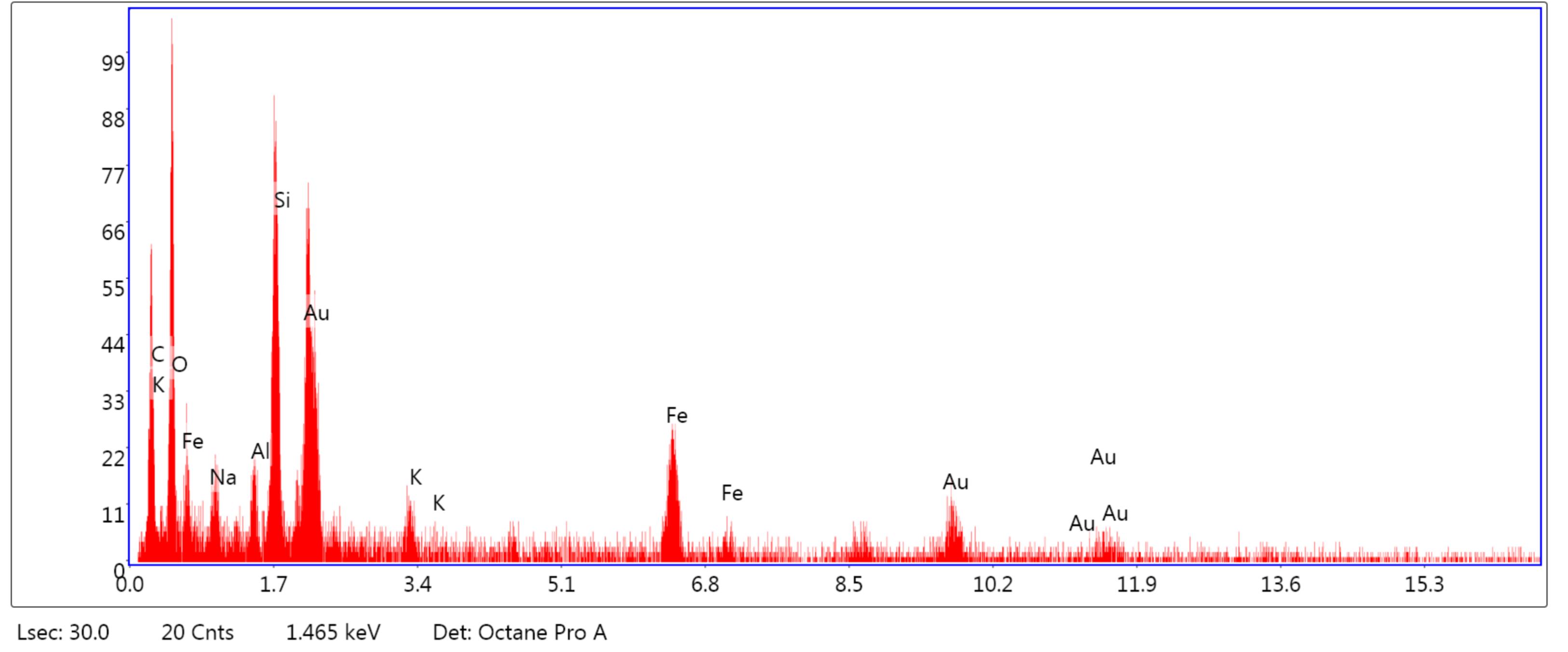

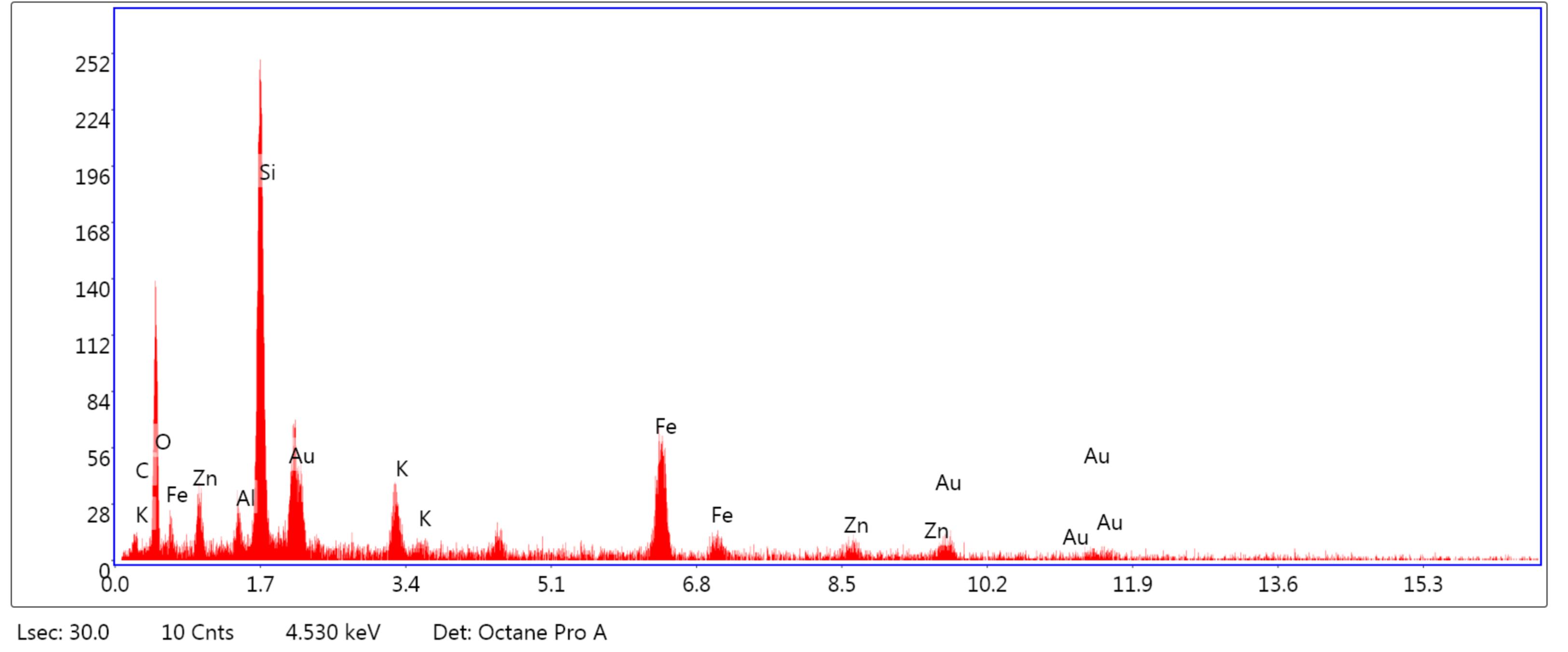

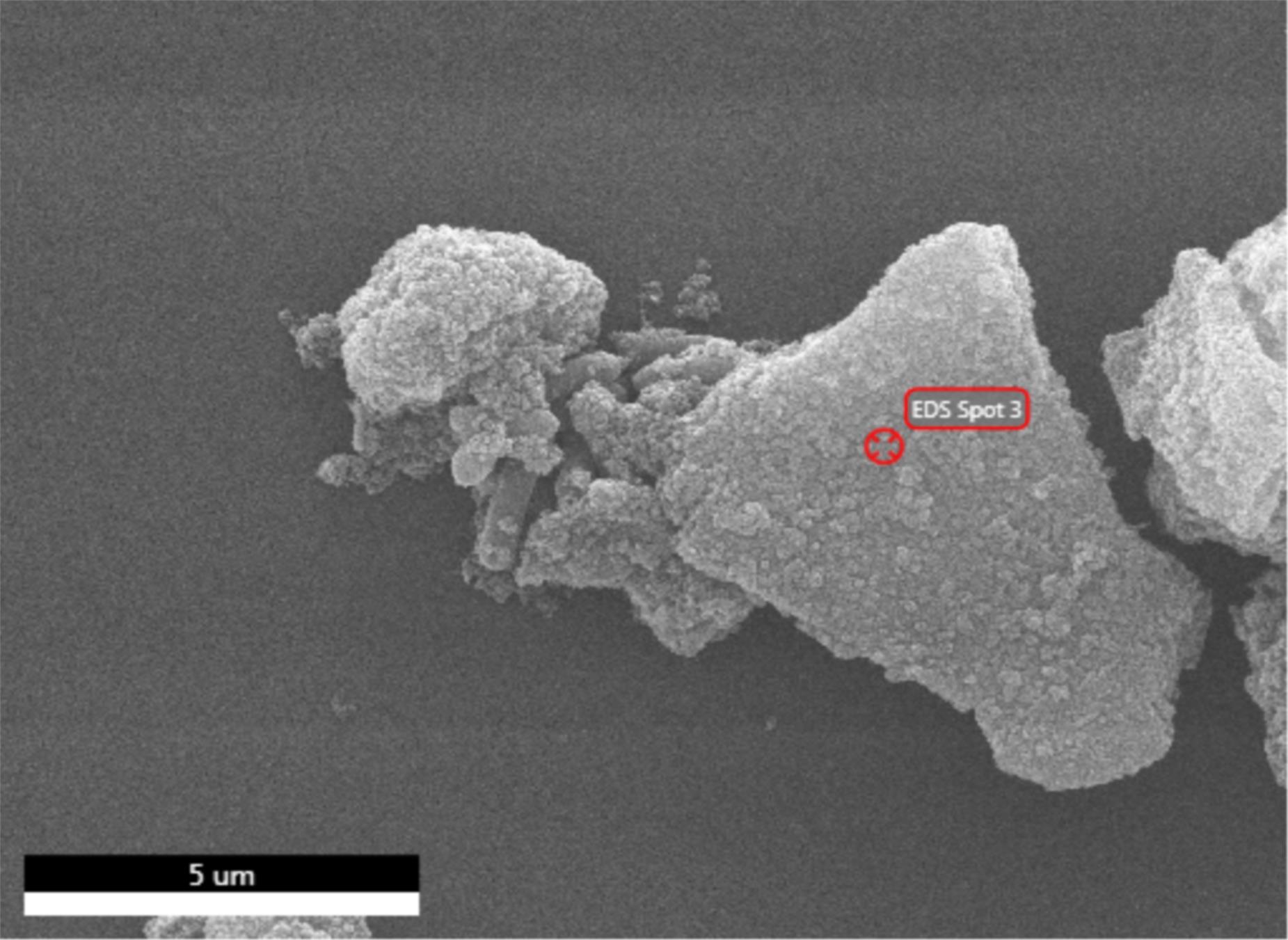


1.25 gFe/L

2.5 gFe/L

**Figure S2**. SEM micrographs and EDS analyses of samples taken from the microcosms amended without magnetite (upper graph), 1.25 gFe/L of magnetite (middle graph), and 2.5 gFe/L of magnetite (lower graph).

Table S1.

Taxonomic assignment and calculations of taxa abundances for the 16S rRNA reads of both Archaea and Bacteria were performed using EMU v3.4.5 (10.1038/s41592-022-01520-4) against the Silva NR99 database v138.2.

*Archaea*

|  | **magnetite concentration (gFe/L)** | | |  |  |  |  |  |  |  |  |  |
| --- | --- | --- | --- | --- | --- | --- | --- | --- | --- | --- | --- | --- |
|  | **0** | **1.25** | **2.5** | **Superkingdom** | **Phylum** | **Class** | **Order** | **Family** | **Genus** | **Species** | **unique** | **.Taxon** |
| *Methanobrevibacter arboriphilus* | 66.43% | 64.32% | 56.07% | Archaea | Methanobacteriota | Methanobacteria | Methanobacteriales | Methanobacteriaceae | Methanobrevibacter | Methanobrevibacter arboriphilus | Methanobrevibacter arboriphilus | Methanobrevibacter arboriphilus |
| *Methanobacterium formicicum* | 30.64% | 35.20% | 43.39% | Archaea | Methanobacteriota | Methanobacteria | Methanobacteriales | Methanobacteriaceae | Methanobacterium | Methanobacterium formicicum | Methanobacterium formicicum | Methanobacterium formicicum |
| Other | 2.93% | 0.49% | 0.54% | Archaea | Methanobacteriota | Methanobacteria | Methanobacteriales | Methanobacteriaceae | Methanobacterium | Other | Other | Other |

*Bacteria*

|  | **magnetite concentration (gFe/L)** | | |  |  |  |  |  |  |  |  |  |
| --- | --- | --- | --- | --- | --- | --- | --- | --- | --- | --- | --- | --- |
|  | **0** | **1.25** | **2.5** | **Superkingdom** | **Phylum** | **Class** | **Order** | **Family** | **Genus** | **Species** | **unique** | **.Taxon** |
| *Sporomusa paucivorans* | 45.18% | 30.95% | 0.00% | Bacteria | Bacillota | Negativicutes | Veillonellales-Selenomonadales | Sporomusaceae | Sporomusa | Sporomusa paucivorans | Sporomusa paucivorans | Sporomusa paucivorans |
| *Sporomusa sphaeroides* | 16.71% | 11.31% | 0.00% | Bacteria | Bacillota | Negativicutes | Veillonellales-Selenomonadales | Sporomusaceae | Sporomusa | Sporomusa sphaeroides | Sporomusa sphaeroides | Sporomusa sphaeroides |
| *Paracoccus versutus* | 0.03% | 18.71% | 29.66% | Bacteria | Pseudomonadota | Alphaproteobacteria | Rhodobacterales | Paracoccaceae | Paracoccus | Paracoccus versutus | Paracoccus versutus | Paracoccus versutus |
| *Paracoccus sp. R-24665* | 0.00% | 3.01% | 29.60% | Bacteria | Pseudomonadota | Alphaproteobacteria | Rhodobacterales | Paracoccaceae | Paracoccus | Paracoccus sp. R-24665 | Paracoccus sp. R-24665 | Paracoccus sp. R-24665 |
| *Thauera sp. R-24450* | 0.01% | 2.14% | 5.79% | Bacteria | Pseudomonadota | Gammaproteobacteria | Burkholderiales | Rhodocyclaceae | Thauera | Thauera sp. R-24450 | Thauera sp. R-24450 | Thauera sp. R-24450 |
| *Thauera humireducens* | 0.00% | 1.99% | 4.01% | Bacteria | Pseudomonadota | Gammaproteobacteria | Burkholderiales | Rhodocyclaceae | Thauera | Thauera humireducens | Thauera humireducens | Thauera humireducens |
| *Acetobacterium sp. ROME195Asa* | 13.03% | 6.25% | 0.93% | Bacteria | Bacillota | Clostridia | Eubacteriales | Eubacteriaceae | Acetobacterium | bacterium ROME195Asa (Acetobacterium) | bacterium ROME195Asa (Acetobacterium) | bacterium ROME195Asa (Acetobacterium) |
| *Azospira oryzae* | 4.26% | 0.78% | 1.42% | Bacteria | Pseudomonadota | Gammaproteobacteria | Burkholderiales | Rhodocyclaceae | Azospira | Azospira oryzae | Azospira oryzae | Azospira oryzae |
| *Arcobacter cibarius* | 5.01% | 0.31% | 0.03% | Bacteria | Campylobacterota | Campylobacteria | Campylobacterales | Arcobacteraceae | Arcobacter | Arcobacter cibarius | Arcobacter cibarius | Arcobacter cibarius |
| *Stenotrophomonas acidaminiphila* | 0.68% | 1.95% | 4.19% | Bacteria | Pseudomonadota | Gammaproteobacteria | Lysobacterales | Lysobacteraceae | Stenotrophomonas | Stenotrophomonas acidaminiphila | Stenotrophomonas acidaminiphila | Stenotrophomonas acidaminiphila |
| *Other* | 15.08% | 22.62% | 24.31% | Bacteria | Bacillota | Clostridia | Eubacteriales | Eubacteriaceae | Acetobacterium | Other | Other | Other |

3
